# Innovations in Refractory Melasma: Mechanism-Based Management and Emerging Therapeutic Strategies

**DOI:** 10.7759/cureus.107411

**Published:** 2026-04-20

**Authors:** Sofía Laguna Rocafort, Jeevan Rivera-Díaz, Ingrid M Bonilla, Jose Rabelo-Cartagena

**Affiliations:** 1 School of Medicine, Universidad Central del Caribe, Bayamón, PRI; 2 Research and Development, Veterans Affairs Caribbean Healthcare System, San Juan, PRI; 3 Dermatology, Veteran Affairs Caribbean Healthcare System, San Juan, PRI

**Keywords:** cholasma, hormonal pigmentation, hyperpigmentation, melanin, melasma

## Abstract

Melasma is a common, chronic, acquired disorder characterized by hyperpigmentation, predominantly affecting women. Although its exact pathophysiology is unknown, multiple factors affect its pathogenic process since it’s considered a multifactorial disease. This review provides an updated narrative synthesis of the epidemiology, pathophysiology, diagnostic approaches, and current management strategies for melasma, with emphasis on emerging therapeutic modalities, including approaches for refractory disease, and clinically relevant insights.

This narrative review was conducted following the Scale for the Assessment of Narrative Review Articles (SANRA) to ensure methodological rigor. A comprehensive literature search was performed across multiple databases, focusing on peer-reviewed studies addressing melasma pathogenesis, diagnosis, and treatment. The findings were synthesized qualitatively.

The pathogenesis of melasma involves complex interactions between ultraviolet radiation, hormonal modulation, dermal remodeling, inflammation, mast-cell activity, and stem cell signaling. Its diagnosis is primarily clinical and may be supported by Wood’s lamp examination or dermoscopy. Management of melasma requires a multimodal approach, with strict photoprotection as the foundation. Therapeutic options include topical depigmenting agents, systemic therapies, chemical peels, microneedling, laser and light-based treatments, and platelet-rich plasma, and emerging combination and drug delivery strategies for refractory melasma, selected based on disease severity, skin type, and risk of recurrence.

Melasma remains a challenging and recurrent pigmentary disorder. Individualized, combination-based treatment strategies grounded in photoprotection and informed by evolving pathogenic insights offer the best potential for sustained clinical improvement, particularly in patients with refractory disease. Continued research is essential to optimize long-term outcomes and quality of life.

## Introduction and background

**Figure 1:**
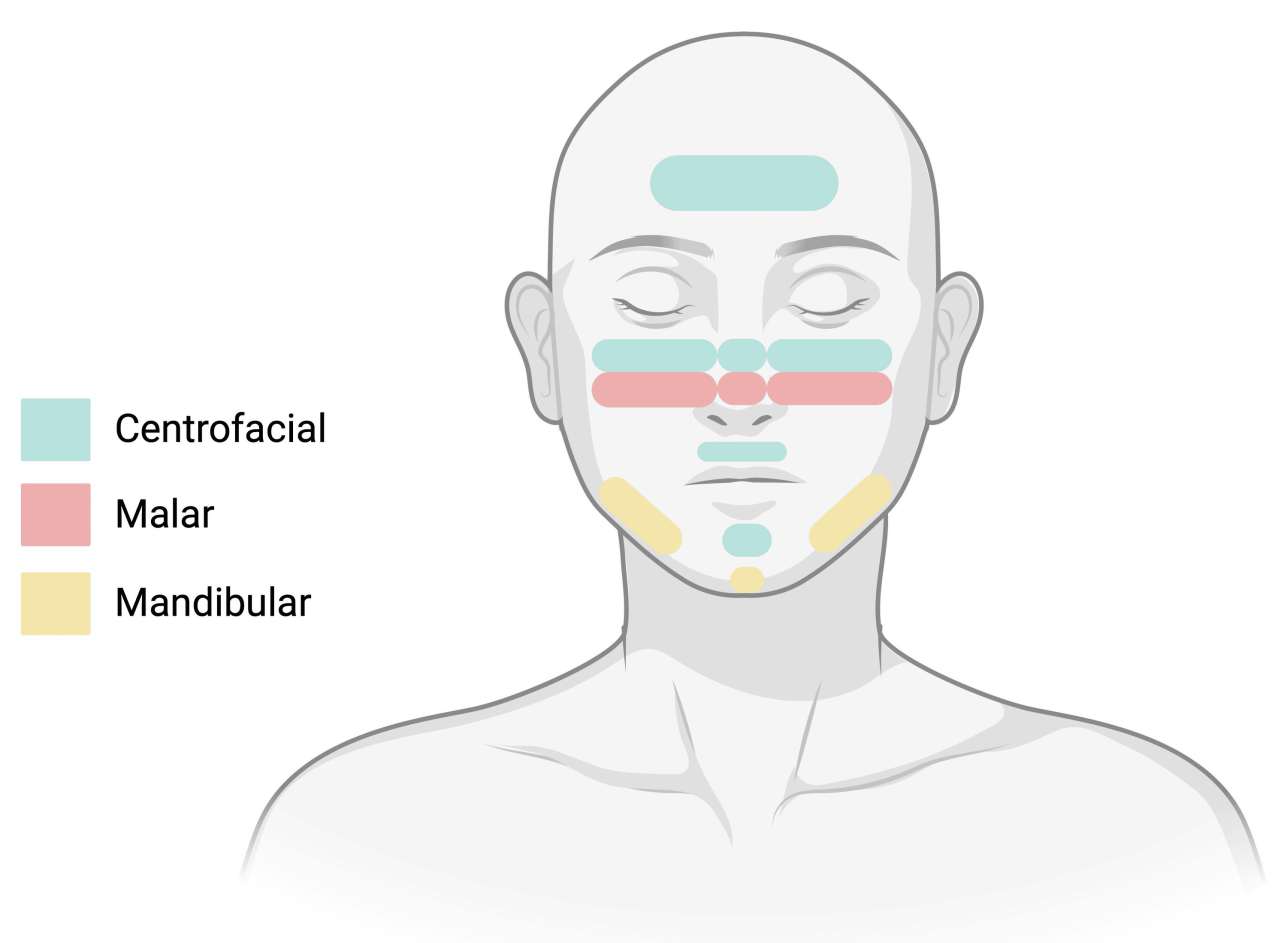
Pattern distribution of melasma. This diagram illustrates the common facial distribution patterns of melasma. The centrofacial pattern (blue) involves the forehead, cheeks, nose, upper lip, and chin and represents the most frequent presentation. The malar pattern (pink) is limited to the cheeks and nasal bridge. The mandibular pattern (yellow) affects the jawline and chin. These patterns may occur individually or in combination. Created in BioRender. Rivera, J., Pattern distribution of melasma (2026). https://www.biorender.com/citation/figure-1-pattern-distribution-of-melasma

Melasma, also known as chloasma, is a common, chronic, acquired hyperpigmentation disorder that predominantly affects women [1]. It presents as symmetric light-to-dark brown macules and patches with irregular borders, mainly on sun-exposed facial areas, with a reported prevalence of 8%-40% depending on ethnicity, and higher in darker-skinned females (typically Asian and Hispanic women with Fitzpatrick skin types III-V) [1-3]. While the exact cause is unknown, triggers include UV radiation, hormonal and genetic factors, hypothyroidism, medications, certain cosmetics, and psychological stress [1]. Melasma is classified by facial distribution into three subtypes: centrofacial (most common), malar, and mandibular (Figure 1), and by pigment location: epidermal melasma is brown with sharp margins, while dermal melasma is gray-brown with poorly defined borders [4].

Although facial hyperpigmentation is painless, it affects appearance and quality of life [1]. Persistent psychological stress from melasma can lead to depression and may worsen the condition by altering melanogenesis through local hypothalamic-pituitary-adrenal (HPA) axis responses [1]. Depression has been associated with increased levels of pro-opiomelanocortin-derived peptides, which may increase melanin production in melanocytes [1].

Understanding the variable pathogenesis and recurrence of melasma helps clinicians guide personalized therapies. However, some patients develop refractory melasma, which is characterized by persistent or recurrent pigmentation despite conventional therapies, highlighting the need for multimodal and emerging treatment approaches [3,4]. This review covers recent contributors and treatment approaches, including pharmacologic, non-pharmacologic, and investigational options.

The primary objective of this narrative review is to synthesize current evidence on the epidemiology, pathophysiology, and management of refractory melasma. Specifically, this review aims to summarize recent advances in understanding the multifactorial mechanisms underlying melasma, including photoaging, hormonal modulation, dermal inflammation, and melanocyte dysregulation. In addition, we review current pharmacologic, procedural, and adjunctive therapeutic strategies, highlighting their clinical applicability, safety considerations, and limitations.

A secondary objective is to integrate available evidence into a clinically relevant framework that supports individualized, multimodal management approaches and identifies gaps in knowledge to guide future research.

**Figure 2:**
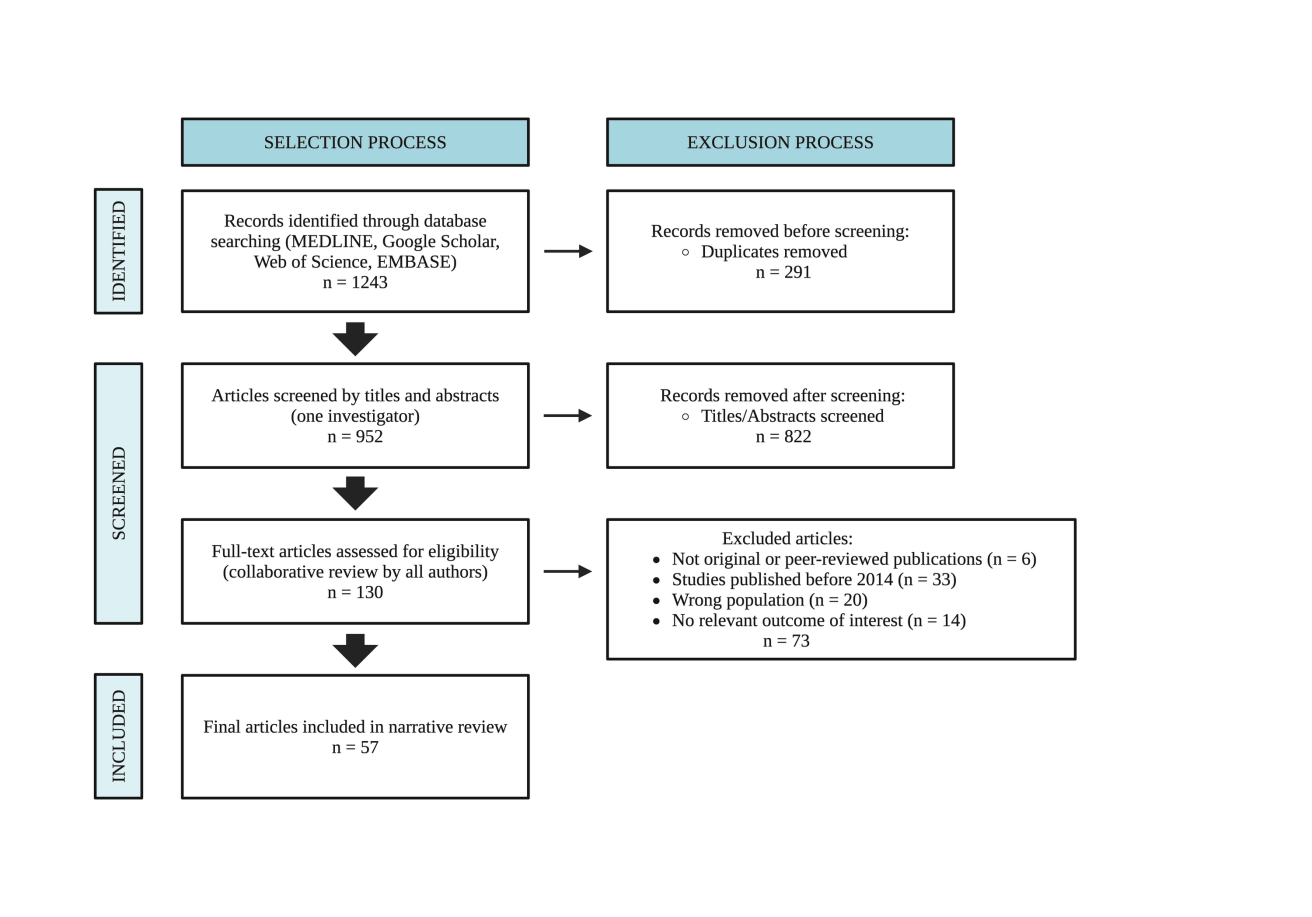
Literature Identification and Screening Process. A total of 1,243 records were initially identified through database searches (MEDLINE, Google Scholar, Web of Science, and Embase) published between July 2014 and September 2025. Following duplicate removal and sequential screening, 57 studies were included in this narrative review in accordance with SANRA quality criteria. Created in BioRender. Rivera, J., Literature identification and screening process (2026). https://BioRender.com/a5tlls6 SANRA: Scale for the Assessment of Narrative Review Articles

This narrative review followed the Scale for the Assessment of Narrative Review Articles (SANRA) to ensure methodological quality and transparent reporting [5]. A comprehensive search was conducted in MEDLINE, Google Scholar, Web of Science, and Embase for English-language studies published between July 2014 and September 2025. Search terms included “melasma”, “chloasma”, “hyperpigmentation”, “treatment”, and “refractory”. Boolean operators (AND/OR) were used to broaden or narrow the results depending on the topic being searched. Eligible articles included peer-reviewed original studies, reviews, and case reports on the epidemiology, pathophysiology, diagnosis, or treatment of melasma. Exclusion criteria included non-peer-reviewed material, non-English publications, studies published before 2014, studies evaluating pigmentary disorders other than melasma and articles that did not address outcomes relevant to melasma epidemiology, pathophysiology, diagnosis, or treatment. After screening and deduplication, 57 publications were included. Reasons for exclusion at the full-text stage were categorized into four groups: wrong population, no relevant outcome of interest, not original or peer-reviewed evidence, and studies published outside the predefined search period. Data were categorized into epidemiology, pathophysiology, diagnosis, and management, and qualitatively synthesized. Potential selection bias was mitigated using multiple databases and independent reference cross-checking (Figure 2). 

## Review

The incidence of melasma is influenced by UV exposure, ethnicity, and hormonal factors. The prevalence of melasma is 0.18% in the United States [6]. Predominantly affecting women with Fitzpatrick skin types III-V, it accounts for 90%-95% of the cases [1,7]. The female-to-male ratio is about 19:1, although in Puerto Rico, men constitute approximately 10% of the patients [7]. Most common between ages 30 and 50, melasma correlates with reproductive and hormonal changes and tends to decrease post-menopause [7]. Higher prevalence is noted in Hispanic and Asian populations, about threefold higher than in white and Black populations [6,7]. In a study of 2,000 Black dermatology patients in Washington, DC, melasma was the second most frequent pigmentary disorder after post-inflammatory hyperpigmentation [7]. Hormonal influences play a significant role, with pregnancy and oral contraceptives triggering melasma in 40%-50% of women, and the condition shows a geographic variation from 10.7% in Brazil to 46% in Pakistan [7]. Between 8% and 34% of women on combined oral contraceptives develop melasma, which has also been reported with post-hormone replacement therapy [7]. Despite advances, the natural history and triggers of melasma remain incompletely understood.

A 2024 study reported hypertension and hormonal contraception as common comorbidities among patients with melasma [8]. In this database analysis of 41,283 patients, hypertension was present in 25% of the melasma cohort, while hormonal contraception was reported in 24% of patients [8]. Rosacea, atopic dermatitis, lupus, history of skin cancer, and internal malignancy also showed elevated odds ratios for melasma development [9]. A 2023 meta-analysis estimated the prevalence of depression at 43.4% among melasma patients, about 12 times the global estimate (3.4%). Patients with melasma had a higher depression risk than controls, as depression may elevate cortisol and pro-opiomelanocortin, potentially increasing melanogenesis [1].

The exact pathophysiology of melasma is unknown but multifactorial [1,9]. Melasma involves increased melanin production with ultraviolet (UV) radiation upregulating melanocyte-stimulating hormone (MSH) receptors, enhancing melanin synthesis [3,4]. Melanogenesis converts tyrosine to melanin via tyrosinase and other enzymes, driving visible pigmentation and photoprotection [3,4]. Chronic sun exposure leads to solar elastosis, with the affected skin showing thicker, fragmented elastic fibers. UVB promotes melanocyte activity via growth factors, cytokines, and increases in keratinocyte plasmin, arachidonic acid, and α-MSH levels [3,4,9]. High-energy visible light (HEVL) and UVA1 reinforce hyperpigmentation, especially in darker skin, through opsin 3 signaling. Mast cells in elastotic areas induce elastin production by fibroblasts, contributing to photoaging and basement membrane (BM) disruption [3,4,9]. Estrogen contributes to the higher prevalence of melasma in post-pubertal women, pregnant women, and oral contraceptive users. Hormonal imbalance may also play a role in men. Increased progesterone receptors in the epidermis and estrogen receptors in the dermis increase melanin production through tyrosinase and microphthalmia-associated transcription factor (MITF) pathways [3]. Chronic UV exposure contributes to dermal remodeling and structural alterations that play a central role in melasma pathogenesis. One of the key changes described is the disruption of the BM, which normally separates the epidermis from the dermis [4]. The disruption of BM is characterized by vacuolar degeneration of basal cells and focal BM changes. Chronic UV exposure increases the expression of matrix metalloproteinase (MMP)-2, degrading type IV/VI collagen, allowing melanocyte descent into the dermis and contributing to pigmentation. This degradation may facilitate the transfer of dermal factors, perpetuating hyperpigmentation [4]. In addition, increased mast cell numbers have been observed in melasma lesions. Mast cells release histamine, which binds to H2 receptors on melanocytes and promotes melanogenesis [3]. UV-induced mast cell tryptase activates MMPs that degrade collagen IV, promoting solar elastosis. Granzyme B from mast cells further injures the extracellular matrix, driving hypervascularization through fibroblast growth factor-2, vascular endothelial growth factor (VEGF), and transforming growth factor beta (TGF-β) [3]. Prolonged UV exposure also induces dermal inflammation, with fibroblasts secreting stem cell factor (SCF), which diffuses into the epidermis to promote melanogenesis. Upregulated c-Kit receptors and SCF-c-Kit signaling activate tyrosine kinase pathways, while elevated cyclooxygenase-2 (COX-2) and prostaglandins further stimulate melanocytes [3]. The overexpression of SCF during hyperpigmentation suggests that this pathway may represent a potential therapeutic target [10].

Understanding the multifactorial pathogenesis of melasma, including UV-induced dermal remodeling, inflammatory mediators, vascular changes, and melanocyte activation, helps explain the rationale behind current therapeutic approaches, many of which target these pathways through photoprotection, inhibition of melanogenesis, anti-inflammatory effects, and dermal remodeling. 

The diagnosis of melasma is primarily clinical, characterized by light-to-dark-brown macules on sun-exposed areas, typically in centrofacial, malar, or mandibular patterns [4,11]. Wood’s lamp helps identify epidermal, dermal, or mixed pigmentation: epidermal lesions enhance, dermal do not, and mixed patterns show both [12,13]. Dermoscopy aids diagnosis, classification, severity scoring, and treatment monitoring, showing pigmented dots, globules, increased vascularity, and telangiectasia [12,14]. Histopathology, reserved for uncertain cases, demonstrates increased melanin deposition within basal and suprabasal keratinocytes of the epidermis, along with BM disruption, pendulous melanocytes, melanophages, increased mast cells, SCF and c-Kit expression, solar elastosis, and neovascularization [15]. Severity is assessed with the Melasma Area and Severity Index (MASI) and the modified MASI (mMASI), improving interobserver reliability through regional weighting [12]. Hormonal evaluation, though not indicated for all patients, may include follicle-stimulating hormone (FSH), luteinizing hormone (LH), melanocyte-stimulating hormone (MSH), thyroid-stimulating hormone (TSH), progesterone, and prolactin if endocrine abnormalities are suspected [11].

**Figure 3:**
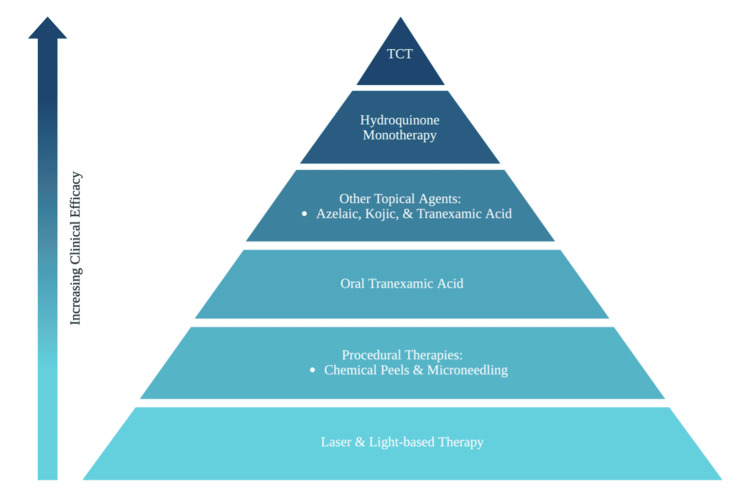
Hierarchy of Effectiveness in Melasma Treatment. This pyramid illustrates increasing treatment effectiveness for melasma from base to apex. Laser and light-based therapies and procedural treatments (chemical peels, microneedling) provide adjunctive benefit, while oral tranexamic acid and topical depigmenting agents (azelaic, kojic, and tranexamic acid) target melanogenesis. Hydroquinone monotherapy is a potent topical treatment, and triple combination therapy (TCT) represents the most effective approach due to its synergistic mechanisms. Created in BioRender. Rivera, J. Hierarchy effectiveness and melasma treatment (2026). https://BioRender.com/hdyjpwy

Effective melasma management requires a multimodal approach grounded in strict photoprotection. Treatment selection depends on disease severity, pigment depth, skin phototype, and prior treatment response. Because melasma is often refractory to treatment, combination strategies are frequently required [12]. Combining topical agents and procedures achieves faster improvement, fewer adverse effects, and better adherence compared to monotherapy [12]. Effective combination therapies include topical depigmenting agents such as hydroquinone and triple combination cream with chemical peels, laser-based devices (e.g., Q-switched Nd:YAG laser, intense pulsed light), microneedling, platelet-rich plasma (PRP), or systemic agents like oral tranexamic acid. These regimens yield greater reductions in MASI scores, enhancing efficacy and occasionally reducing adverse effects like post-inflammatory hyperpigmentation (PIH) [13-17]. Tailoring treatments to individual patient characteristics, emphasizing rigorous photoprotection, and periodic reassessment are crucial for optimal outcomes and minimizing recurrence [18]. Figure 3 summarizes the relative effectiveness of available therapies. Vigilance is required for potential adverse effects, especially with aggressive procedures. More research is necessary to clarify the long-term safety and optimal combination strategies [13,17,19].

Among current treatments, the highest-quality evidence supports triple combination therapy (hydroquinone, tretinoin, and corticosteroid) and oral tranexamic acid (TA), followed by chemical peels and microneedling as effective adjuncts. Platelet-rich plasma, thiamidol, cysteamine, and device-based approaches are supported mainly by small trials or case series, warranting cautious interpretation.

Hydroquinone, especially at 4%, remains a highly effective depigmenting agent by inhibiting tyrosinase to reduce melanin production. Its common side effects include irritation, with rare cases of exogenous ochronosis [19-23]. Retinoids enhance hydroquinone efficacy and act synergistically with sunscreen, inhibiting tyrosinase transcription, but are contraindicated during pregnancy [17,18]. Niacinamide offers similar improvement to 4% hydroquinone with fewer side effects, often used at 4%-5% concentration with sunscreen or retinoids [24].

Vitamin C has demonstrated improvement in melasma severity in several studies, particularly when combined with microneedling or fractional CO₂ laser [25-28]. Cysteamine, a naturally occurring molecule, offers a good safety profile and efficacy comparable to hydroquinone, without typical side effects [28,29]. Azelaic acid (AZA) is effective as monotherapy and suitable for those intolerant to hydroquinone or combined with oral TA or peels for severe cases [18,30]. Kojic acid is effective on its own but yields better results when combined with hydroquinone [19,31]. Arbutin has been reported to improve hyperpigmentation in patients with melasma [32]. Similarly, thiamidol, a novel tyrosinase inhibitor, has demonstrated improvement in melasma in up to 79% of treated patients in clinical studies and may be a suitable option for individuals intolerant to hydroquinone [17,33].

Photoprotection is crucial to prevent new lesions and worsening of existing ones. Recommended measures include minimizing sun exposure, using protective clothing, and consistently applying broad-spectrum UVA/UVB sunscreens with a sun protection factor (SPF) >30. Sunscreens containing iron oxide are particularly beneficial because they provide protection against visible light, an important trigger for melasma exacerbation and recurrence. Long-term photoprotection reduces relapse risk [9,12,17].

Systemic treatments provide additional options for melasma. Tranexamic acid, an antifibrinolytic that inhibits melanin synthesis, is effective and can be administered orally, topically, by mesotherapy, or microneedling, though oral use shows the best outcomes [17]. It requires monitoring for thrombotic risks and recurrence can occur post-cessation [34,35]. Melatonin shows promise as a combination therapy with slight MASI improvement, though further study is needed [36,37]. Ongoing research investigates systemic agents such as procyanidin, pycnogenol, polypodium leucotomos, synbiotics, and finasteride, which show potential for use in combination or maintenance therapy, although current evidence remains limited [19,30,37].

Procedures should be tailored to melasma type and skin phototype. Epidermal melasma responds well to topical agents and superficial peels, whereas dermal or mixed types benefit more from microneedling or low-fluence lasers. In Fitzpatrick IV-VI skin, procedures must be approached cautiously due to increased risks of PIH and scarring [12,18,38-40].

Microneedling uses fine needles delivered through stamping devices, rollers, or automated pens and has been shown to be effective in the treatment of melasma [41-43]. Most studies evaluating therapeutic outcomes involve clinical microneedling performed at greater needle depths (generally ≥0.5-1.5 mm) to induce dermal remodeling and enhance transdermal drug delivery [41,42]. In contrast, superficial cosmetic microneedling (<0.5 mm) may be used primarily to improve topical drug absorption depending on the therapeutic objective [41]. Histologic changes following microneedling have been reported within the first week after treatment [41,42]. Reported adverse effects include transient erythema, burning, pain, swelling, and bruising, with recovery time and risk profile varying according to needle depth and treatment intensity [13,41-43].

Chemical peels are popular for their rapid response, safety, and cost-effectiveness, often as second-line treatments after depigmenting agents [12]. They are particularly effective for recent-onset melasma, relying heavily on proper patient selection, meticulous pre- and post-procedure care, and thorough counseling. Medium and deep peels are generally contraindicated in darker skin due to risks of PIH, dyspigmentation, and scarring, and should be avoided during pregnancy [12]. Glycolic acid (GA), the most studied peel, disrupts corneocyte adhesion and inhibits tyrosinase. Despite its benefits, GA poses a PIH risk in darker skin [18,38,44]. Studies show 30% GA and 15% trichloroacetic acid (TCA) both reduce MASI effectively, outperforming 92% lactic acid (LA). However, TCA is less favored due to higher risks of scarring and dyschromia in darker phototypes. LA 92% is comparable to Jessner’s solution, offering a safe alternative [18,38,44,45].

Q-switched Nd:YAG laser (QSNYL) is preferred for dermal/mixed melasma due to its 532/1064-nm wavelengths, allowing deep penetration and selective photothermolysis of melanosomes, with the 1064-nm wavelength being optimal for dermal lesions [45,46]. Picosecond 1064-nm (PSNY) lasers achieve comparable MASI reductions with fewer adverse effects [47]. Despite initial improvements, relapse rates after laser therapy are high, with common adverse effects including mottled hypopigmentation, punctate leukoderma, and rebound hyperpigmentation. Therefore, lasers are primarily reserved for refractory cases [18,46].

PRP has been investigated as an adjunctive treatment for melasma, with clinical studies reporting improvement in pigmentation and MASI scores following treatment [48-51]. Studies show PRP can outperform TA in reducing MASI. Combining topical 5% TA (liposomal) with autologous PRP injections yields superior results compared to TA alone. A meta-analysis of 10 studies (n=395) found PRP, particularly with microneedling, significantly reduced severity (mMASI -1.18) with minimal adverse events [48-51].

Refractory melasma remains one of the most challenging presentations of this pigmentary disorder, characterized by inadequate response to conventional therapies and frequent relapse after treatment discontinuation. Although no universally accepted definition exists, refractory melasma has been broadly described as a persistent disease despite multiple therapeutic interventions such as topical depigmenting agents, chemical peels, laser therapies, or microdermabrasion over extended treatment periods [52]. These patients often require escalation strategies that address the multifactorial pathogenesis of melasma and the limitations of traditional monotherapy.

One emerging systemic approach involves the use of oral TA, which has gained attention due to its antifibrinolytic activity and its ability to interfere with melanogenesis through the inhibition of the plasminogen-plasmin pathway [53]. In a recent randomized clinical trial comparing oral and topical TA formulations, both treatment modalities demonstrated significant reductions in MASI scores after 12 weeks of therapy, highlighting the therapeutic potential of TA as a component of melasma management [53]. These findings support the growing role of TA as an adjunctive therapy in patients who show suboptimal response to topical regimens alone [53].

Advances in drug delivery techniques have also contributed to the development of innovative therapeutic approaches. Microneedling-assisted transdermal delivery of pharmacologic agents has demonstrated promising outcomes in recent studies [54]. In a split-face comparative study, microneedling combined with topical metformin resulted in greater improvement in hemi-MASI scores compared with microneedling combined with topical vitamin C [26]. The authors proposed that metformin may exert melanin-reducing effects, potentially through modulation of melanogenesis pathways, while microneedling enhances dermal drug penetration. These findings suggest that enhanced transdermal delivery strategies may represent an effective option for patients with persistent disease [26].

Combination topical therapies targeting multiple mechanisms of melanogenesis have also shown potential in resistant cases. A prospective pilot study evaluating a formulation containing topical TA (2%) combined with vitamin C (2%) demonstrated significant reductions in MASI scores over an eight-week treatment period in patients with resistant melasma [54]. Improvements were also observed in patient-reported quality of life measures and physician global assessments, suggesting that multimodal topical regimens may offer a well-tolerated alternative for patients who do not respond adequately to conventional bleaching agents [54].

Procedural approaches combined with targeted pharmacologic therapy have further expanded treatment options. In a prospective split-face study evaluating fractional carbon dioxide laser-assisted delivery of TA versus ascorbic acid, trans-epidermal delivery following laser treatment produced clinical improvement in melasma lesions [25]. Notably, the TA-treated side demonstrated greater improvement compared with the ascorbic acid-treated side, suggesting that laser-assisted drug delivery may enhance therapeutic outcomes by improving penetration of active agents into the epidermis and dermis [25].

The need for multimodal therapeutic strategies is further supported by broader reviews of melasma management. Recent literature highlights that commonly used therapies are often limited by incomplete clinical effectiveness, relapse after treatment discontinuation, and potential adverse effects [55]. As a result, emerging research has focused on improving drug delivery systems, including nanotechnology-based formulations designed to enhance skin penetration and reduce systemic exposure [56]. These approaches aim to optimize the therapeutic index of topical agents while minimizing adverse effects [56].

Finally, comparative analyses of available therapies underscore the variability in treatment outcomes and the benefits of combination regimens. A network meta-analysis evaluating multiple melasma treatments reported that combination therapies frequently demonstrate greater efficacy than single-agent approaches, emphasizing the importance of individualized therapeutic strategies that target multiple pathogenic pathways simultaneously [23]. Collectively, these findings support a paradigm shift in melasma management toward multimodal, mechanism-informed treatment strategies, particularly in patients with refractory disease [57].

For epidermal melasma, superficial chemical peels (e.g., glycolic acid 20%-70%) and microneedling are effective due to the superficial pigment [19,20,23,44]. Dermal or mixed melasma responds less effectively to peels because of deeper pigment; thus, cautious use of laser and light-based therapies (e.g., low-fluence Q-switched Nd:YAG, ablative fractional laser) is recommended due to higher risks of PIH and recurrence [19,20,23,44].

In darker phototypes (IV-VI), the risk of PIH and other adverse effects increases. Gentle procedures like superficial peels (glycolic acid ≤30%) and minimal trauma microneedling are preferred. Aggressive lasers and deep peels should be avoided. If laser therapy is necessary, it should employ lower energy settings and longer intervals between sessions. Lighter phototypes (I-III) can tolerate deeper peels and higher energy lasers, but photoprotection is essential for all to prevent recurrent and minimize adverse effects [1,4,5,8,20,29,30].

TA is generally well tolerated and can reduce PIH, especially when combined with procedures like lasers or microneedling, particularly in patients with skin of color [31]. Common side effects include mild gastrointestinal upset, hypomenorrhea, and skin irritation, with a low but existent risk of thrombotic events. Large studies report rare thromboembolic events, emphasizing the need for prior screening for thrombotic risks. The use of TA should be avoided in patients with active thromboembolic disease, known thrombophilia, or during high-risk periods like pregnancy or estrogen therapy [32-38].

Chemical peels are contraindicated in cases of active skin infections, open wounds, allergies to the peeling agent, and recent isotretinoin use due to risks of scarring and delayed healing [39,40]. Caution is advised for darker skin due to the risk of PIH, history of keloids, pregnancy, sensitive skin, recent sunburn, and unrealistic outcome expectations [17,39].

Absolute contraindications for laser therapy include active skin infections, photosensitivity disorders, recent isotretinoin use, pregnancy (with certain devices), and a history of seizures (for light-based therapies). Relative contraindications include darker phototypes (PIH/hypopigmentation risk), dermal/mixed melasma (poor response), history of keloids, recent tanning, unrealistic expectations, and poor compliance with photoprotection [38,41-43]. Key management strategies have been systematically synthesized and consolidated to facilitate clarity and practical application (Tables 1, 2). 

**Table 1:** Melasma Treatment: Pharmacological Approaches.

Drug Name	Mechanism	Dose	References
Triple Combination Therapy	Retinoids improve the epidermal penetration of HQ and help prevent its oxidation, while the steroid minimizes skin irritation caused by both HQ and retinoids.	2% HQ, 0.025% tretinoin, and 0.01% fluocinolone acetonide	^[18,20]^
Hydroquinone (HQ)	Reversible inhibition of tyrosinase, an enzyme responsible for converting L-3,4-dihydroxyphenylalanine (L-DOPA) into melanin.	4% HQ	^[19,21]^
Retinoid	Retinoids enhance keratinocytes’ metabolism and turnover, reduce melanosome transfer, accelerate melanin loss, and facilitate transepidermal penetration of other topical medication (e.g., enhancing the effectiveness of HQ and AZ).	0.05%-0.1% Tretinoin	^[19,30]^
Niacinamide	Active form of Vitamin B3 that decreases pigmentation by preventing melanosome transfer to keratinocytes.	4%-5%	^[44]^
Vitamin C	Antioxidant that binds to copper to inhibit tyrosinase activity and prevents the oxidative polymerization of melanin intermediates, thereby reducing melanin production in the melanogenesis pathway.	10-20%	^[21,44]^
Cysteamine	Inhibitor of tyrosinase and peroxidase, primary enzymes involved in melanin biosynthesis.	5% cysteamine	^[44]^
Azelaic Acid	Natural dicarboxylic acid that inhibits tyrosinase and suppresses reactive oxygen species involved in the melanogenesis pathway.	20% azelaic acid	^[19,44]^
Kojic acid	Natural fungal metabolite that chelates divalent ions, neutralizes free radicals, and inhibits tyrosinase.	1% KA	^[31,44]^
Arbutin	HQ derivative that competitively inhibits tyrosinase, thereby reducing melanogenesis.	2.51% arbutin	^[44]^
Thiamidol	Tyrosinase inhibitor.	0.2% thiamidol	^[33]^
Tranexamic Acid	Antifibrinolytic agent that indirectly reduces epidermal melanocyte tyrosinase activity by preventing plasminogen binding to keratinocytes, and reducing α-MSH levels.	250mg/day	^[19,22,34]^

**Table 2:** Miscellaneous Approaches. GA: Glycolic acid, LA: lactic acid, TCA: trichloroacetic acid, QSNYL: Q-switched Nd:YAG laser, PSNY: Picosecond 1064-nm

Types	Mechanism	Dose	References
Microneedling	Improves transdermal drug delivery and has been implicated in the activation of genes involved in epidermal differentiation and tissue remodeling, primarily based on vitro studies.	TA (4 mg) applied afterwards mostly	^[41–43]^
Chemical Peels	Accelerate epidermal turnover, promote orderly collagen and elastin deposition in the dermis, enhance phagocytosis of melanin granules, and improve penetration of topical agents.	GA (50%), LA (92%), TCA (15%), Tretinoin (1 or 10%), & Jessner’s solution [resorcinol (14 mg), SA (14 mg), LA (85%/14 mL), and ethyl alcohol (95%/100 mL)]	^[12,38,39]^
Lasers	Deep skin penetration and selective photo-thermolysis of melanosomes.	QSNYL (532 and 1064 nm) and PSNY (1064 nm)	^[40,45-46]^
Platelet-Rich Plasma	May reduce melanin production potentially by modulating extracellular signal-regulated kinase.	Intradermal injection, either alone or adjunct with TA or Q-switched laser	^[48-49,51]^
Melatonin	Modulates melanogenesis through antioxidant and anti-inflammatory effects, including downregulation of tyrosinase activity and inducible nitric oxide synthase (iNOS).	5 mg (Oral)	^[36-37]^

## Conclusions

Melasma, predominantly affecting women, has a significant psychosocial impact. UV exposure, hormonal influences, dermal remodeling, mast-cell mediators, and inflammation sustain hyperpigmentation. Effective management requires continuous photoprotection and multimodal therapy. First-line treatment is triple combination therapy (HQ, retinoid, steroid). Alternatives and adjuncts include AZA, niacinamide, vitamin C, cysteamine, thiamidol, GA chemical peels, microneedling, PRP, and lasers/light devices for refractory cases. Emerging strategies such as combination topical regimens, enhanced drug-delivery techniques, and systemic therapies, including oral TA, may provide additional options for resistant disease. Ongoing research aims to refine SCF-c-Kit and inflammatory pathway targets, optimize regimens, and improve relapse prevention. Addressing mental health is vital. Personalized, sustained treatments with photoprotection and combination-based therapeutic strategies offer the best outcomes and quality of life improvements.
